# *“It’s only fatness, it doesn’t kill”*: a qualitative study on perceptions of weight gain from use of dolutegravir-based regimens in women living with HIV in Uganda

**DOI:** 10.1186/s12905-022-01814-x

**Published:** 2022-06-21

**Authors:** Yussif Alhassan, Adelline Twimukye, Thokozile Malaba, Landon Myer, Catriona Waitt, Mohammed Lamorde, Angela Colbers, Helen Reynolds, Saye Khoo, Miriam Taegtmeyer

**Affiliations:** 1 Department of International Public Health, Liverpool School of Tropical Medicine, Pembroke Place, Liverpool, L3 5QA UK; 2 Infectious Diseases Institute, Makerere University, Kampala, Uganda; 3 School of Public Health and Family Medicine, University of Cape Town, Cape Town, South Africa; 4 Institute of Systems, Molecular and Integrative Biology, University of Liverpool, Liverpool, UK; 5 Radboud University Nijmegen Medical Center, Radboud Institute for Health Sciences, Nijmegen, The Netherlands; 6 Tropical Infectious Diseases Unit, Royal Liverpool University Hospital, Liverpool, UK

**Keywords:** Dolutegravir, Weight gain, Body image, Women, HIV, Uganda

## Abstract

**Background:**

Dolutegravir (DTG)-based regimens have been recommended by the WHO as the preferred first-line and second-line HIV treatment in all populations. Evidence suggests an association with weight gain, particularly among black women. Our study investigated perceptions of weight gain from DTG-based regimen use on body image and adherence of antiretroviral therapy in women living with HIV (WLHIV) in Uganda.

**Methods:**

Between April and June 2021, we conducted semi-structured interviews involving 25 WLHIV (adolescents, women of reproductive potential and post-menopausal women) and 19 healthcare professionals (clinicians, nurses, ART managers and counsellors) purposively selected from HIV clinics in Kampala. The interviews explored perceptions of body weight and image; experiences and management of weight related side effects associated with DTG; and knowledge and communication of DTG-related risks. Data was analysed thematically in NVivo 12 software.

**Results:**

Our findings indicate WLHIV in Uganda commonly disliked thin body size and aspired to gain moderate to high level body weight to improve their body image, social standing and hide their sero-positive status. Both WLHIV and healthcare professionals widely associated weight gain with DTG use, although it was rarely perceived as an adverse event and was unlikely to be reported or to alter medication adherence. Clinical management and pharmacovigilance of DTG-related weight gain were hampered by the limited knowledge of WLHIV of the health risks of being over-weight and obesity; lack of diagnostic equipment and resources; and limited clinical guidance for managing weight gain and associated cardiovascular and metabolic comorbidities.

**Conclusions:**

The study highlights the significance of large body-size in promoting psychosocial wellbeing in WLHIV in Uganda. Although weight gain is recognized as a side effect of DTG, it may be welcomed by some WLHIV. Healthcare professionals should actively talk about and monitor for weight gain and occurrence of associated comorbidities to facilitate timely interventions. Improved supply of diagnostic equipment and support with sufficient guidance for managing weight gain for healthcare professionals in Uganda are recommended.

**Supplementary Information:**

The online version contains supplementary material available at 10.1186/s12905-022-01814-x.

## Background

Dolutegravir (DTG) has superior virologic efficacy, tolerability, safety profile, and limited drug-drug interactions, as compared to alternative antiretroviral therapy regimens for HIV [1, 2]. Since 2016, the World Health Organisation (WHO) has recommended DTG-based regimens as preferred for first-line and second-line treatment for HIV in low-income and middle-income countries [2]. There is growing concern however, that integrase inhibitors, including DTG, are associated with weight gain [3], and related complications, such as diabetes and hypertension [4]. The NAMSAL trial in Cameroon found participants randomised to DTG gained significantly more weight than those in the efavirenz (EFV) arm at 48 weeks of exposure to the treatments [5]. Similar findings have been reported by the ADVANCE trial in South Africa, with antiretroviral therapy (ART)-naïve participants randomised to DTG experiencing more weight gain [6]. A switch study to assess the differential effect of DTG on body weight showed a significant but small weight gain after participants were switched from ritonavir-boosted protease inhibitor–based regimen [7]. While not all ART-induced weight gain is harmful, since it may represent treatment efficacy and return to normal weight, continued weight gain may affect life expectancy and quality of life as patients age and risks of co-morbidities increase [8].

ART-related weight gain has a genetic component and is more pronounced in women, especially post-menopausal women [9, 10], with Black African women more susceptible [3, 11]. There is a rapid rise of overweight and obesity in Africa due to increasing urbanisation and related conventional risk factors [12], which coupled with the high prevalence of HIV and other communicable disease makes the problem particularly intractable. With African women more prone to both HIV infection and obesity [13], they risk being disproportionately affected by ART-related cardiovascular and metabolic diseases.

Weight gain from DTG use may have psychosocial implications for PLHIV, especially among women [14], including altered body image and poor adherence to treatment. Literature on the impacts of altered body image resulting from ART use is divergent. Wagner and Rabkin found psychological wellbeing of PLHIV was affected negatively by increased weight loss [15]. Body dissatisfaction has been linked with depression [16] and symptomatic disease in HIV with resulting implications on treatment and antiretroviral therapy adherence [17]. Corless and colleagues found larger body size correlated with better perceived quality of life [17]. A qualitative study in South Africa found increased body size was perceived as a marker of well-being [18]. Furthermore, two-thirds of adolescent women in a study in Cape Town (South Africa) associated increased body mass with happiness and health [19]. It is envisaged that the attitude of women living with HIV (WLHIV) towards DTG-related weight gain will be influenced by pre-existing body image and size preferences as mediated by socio-cultural norms as well as beliefs about weight-related health risks.

In Uganda, the focus of the current study, national roll-out of DTG-based ART commenced in March 2018, with eligible first-line treatment patients initiated on a fixed-dose combination of TDF/lamivudine (3TC)/DTG (TLD) [20]. Several Ugandan studies have since reported hyperglycaemia [21] and increased appetite and weight gain associated with DTG [22, 23], although evidence on the implications for treatment adherence and psychosocial wellbeing is scant. The Ugandan HIV treatment guidelines recommend weight monitoring and early treatment of complications in patients who initiate DTG. This paper seeks to explore perceptions and experiences of DTG-related weight gain in WLHIV, including potential effects on body image and medication adherence, as well as providers’ experiences of managing weight-gain issues in patients.

## Methods

### Study design

We adopted a cross-sectional descriptive qualitative design because it provides the most potential for exploring participant perspectives and the context in which they live [24]. As the study focuses on participants’ lived experiences it was embedded in an interpretivist paradigm; this emphasises individual subjective meanings and how they are influenced by environment to understand social phenomenon [25]. The perceptions of WLHIV and healthcare workers (HCWs) were explored through interviews to explicate the research questions: how do WLHIV perceive weight gain resulting from the use of DTG-based regimen? How might DTG-based weight gain affect body image and ART medication adherence among WLHIV? What are the main challenges to managing DTG-associated weight gain in health facilities in Uganda?

### Study settings

Study participants were selected from HIV clinics across Kampala, Uganda. The country has a generalized HIV epidemic: there were 1.4 million PLHIV in 2018; women account for 55% of adult PLHIV population [26]; and Kampala has an estimated HIV prevalence rate of 6.9% [27]. Obesity has been on the rise in Uganda since 1995, with women from urban areas and non-poor households more at risk [28]. Uganda runs a vertical HIV care delivery model, with most clinics operating as stand-alone units dedicated to HIV services within general healthcare facilities [22, 29].

### Study population and participant selection

Two participant categories were involved in the study: WLHIV and health care providers routinely attending to ART patients. WLHIV were included to explore user perspectives on weight gain, body image and adherence; the latter to understand the challenges of dealing with weight-related side effects. We focused on women (rather than men) living with HIV due to their increased vulnerability to weight gain and body image challenges [14]. A wide range of HCWs were included to enrich the diversity and complexity of the data (Table 2): pharmacists because they were involved in ART administration and education and were the point of contact for reporting side effects; clinicians and nurses as they were ART prescribers and decisionmakers over regimen changes; and counsellors due to their role in providing treatment literacy. WLHIV participants in three age brackets (18–24 years; 25–49 years; and > 49 years) were included to explore the views and experience of adolescents, women of reproductive potential and post-menopausal women. Although we had set the minimum age for participation to 15 years to capture the views of adolescents, only those aged 19 years and over participated.

Health care providers were sampled purposively [24] based on their specialist knowledge, willingness to participate, and ability to provide consent. WLHIV were also selected purposively, and included if they had used a DTG-based regimen for least 3 months; and had experienced weight gain or other side effects of DTG. WLHIV were approached in-person at routine HIV care and treatment visits. Participating health facilities were drawn from different levels of the Ugandan healthcare system; including two Level 3 sub-county primary health care facilities; one Level 4 district hospital; and one specialist hospital. Sample size was determined by data saturation, deemed to have been reached when no new themes emerged at daily debriefing meetings involving the research team [30].

### Data collection

Data were collected through individual interviews to provide participants with privacy to speak openly due to the sensitive nature of the study topic [24]. The interviews were conducted from April to June 2021 by three experienced Ugandan qualitative researchers, including one of the authors (AT), with extensive knowledge of the local culture, language and healthcare system. They were conducted in-person at the participant’s preferred location, including hospital premises and other safe community locations. Interviews lasted approximately 45 min, were conducted in Luganda or English, audio-recorded and complemented by written notes. Semi-structured topic guides informed the interviews and explored perceptions of body weight and image; experiences and management of DTG side effects; and knowledge and communication of DTG-related risks (Additional file 1: Appendix 1). Questions were piloted and revised iteratively as data collection evolved.

### Data analysis

Interviews were transcribed by a professional typist using a denaturalised approach; transcripts were non-verbatim, with repeated words, pauses and stutters removed [31]. The Luganda transcripts were then translated into English. All transcripts were reviewed for accuracy and completeness. Data were analysed with the assistance of NVivo12 software following the five steps of thematic framework approach: familiarization; identifying a thematic framework; indexing; charting; and mapping and interpretation [32]. Two separate coding frameworks (one for WLHIV and one for HCWs) were inductively developed by AY and AT based on the topic guides and data from 10% of the transcripts. Following an inductive analytic approach, each transcript was read for recurrent ideas; codes assigned to relevant segments of text and similar codes aggregated to form themes [33]. Themes distilled from the two separate frameworks were triangulated to answer the research questions. To improve the quality of the results, transcripts were independently coded, compared and discussed. Emerging findings were discussed among the authors in a consortium meeting and presented in a national health conference in Uganda where feedback was obtained and subsequently integrated into the analysis.. This study is reported in accordance with the Consolidated Criteria for Reporting Qualitative Research (COREQ) [34] (Additional file 2: Appendix 2).

## Results

Forty-four participants took part in the study: 25 WLHIV and 19 HCWs (Tables 1, 2). Participation in the study was high; only 4 of eligible individuals invited failed to participate, with time constraints given as the main reason. Most of the WLHIV participants were young adults and middle-aged women and had received primary or secondary level education. The HCWs were mainly female, with their HIV-related work experience ranging from 3 to 13 years. Important themes of DTG-related weight gain being a welcome (rather than a worrying) side effect and of the barriers to recognising, monitoring and treating weight gain are described below along with illustrative quotes from the data.

**Table 1:** Participant characteristics (Women living with HIV)

	Number	%
Age range		
18—24 years (Younger women)	10	40
25—49 years (Middle-aged women)	10	40
> 49 years (Older women)	5	20
Education		
No education	1	4
Primary	11	44
Secondary/High school	11	44
Post-secondary	2	8
Employment		
Unemployed	7	28
Housewife	2	8
Vendor/trader	2	8
Student	1	4
Volunteer	4	16
Formal sector worker	1	4
Retail business	7	28
Waitress	1	4
Total	25	100

**Table 2:** Participant characteristics (healthcare workers)

	Number	%
Cadre		
Clinician	6	31.5
Nurse/Counsellor	6	31.5
ART manager	4	21.1
Pharmacist	3	15.7
Gender		
Male	8	42.1
Female	11	57.9
Total	19	100

### Weight-gain perceived association with DTG-based regimen

In general, participants felt there were fewer side effects from DTG than other regimens. Health care providers associated DTG with weight gain, hyperglycaemia, and diabetes, with all the HCWs participants reporting they “frequently “encountered such conditions in DTG patients. Of the 25 WLHIV participants initiated or transitioned to DTG-based regimens, 9 reported weight gain; 4 increased appetite; and 2 reported diabetes. Three said they had experienced weight loss and attributed this to reduced appetite following the DTG-based regimen. Additional perceived DTG side effects included reduced libido, joint pain, and insomnia. Most of the WLHIV who had experienced weight gain were unaware it could be due to their medication, and only found out during the interview.

### WLHIV aversion to thinness

The majority of WLHIV participants described their current body size as ‘slim’, attributing it to the distress of their diagnosis and previous ART-related side effects and wanted to regain their normal weight. WLHIV appeared to have a strong aversion towards the thin body figure, mainly because it was commonly associated with serious ill-health and could lead to people suspecting their HIV sero-positive status. Thin people were reported to be widely stereotyped and stigmatised as HIV patients, as noted by these women:

Some people are so slim to the extent that everyone says, "HIV is finishing that one" "see how the clothes are not fitting her". They point fingers at us, …they despise us. We should have bodies that are not slim. (Older woman living with HIV)

Thinness was associated with low self-esteem and a lack of confidence in body appearance which constrained social participation, as noted by this middle-aged WLHIV: *“I want a body that does not embarrass me, that does not scare people, that looks good, one which is not too slim.”* WLHIV participants who experienced weight loss after DTG were displeased and noted it affected confidence and social participation.

That drug caused me problems because those that know me; when they see me now, they talk a lot. Some say, ‘maybe she got HIV, or I got an abortion, or she is using suspicious drugs’. ... I’m even scared of going to the village*.* (Younger woman living with HIV)

### Body size preference

Most WLHIV identified their preferred body size as ‘medium’, commonly described it as “not slim and not too fat”, or “their normal body size prior to the HIV infection”. When probed on how big a ‘medium’ woman was, this appeared to correlate more with clinical categories of overweight and obese than with a body mass index in the healthy range. Moderately large body size was widely perceived to be attractive and associated with prosperity and good health.

I want to have a big body size because even if a fat woman is stressed or does not have money, she remains fat…even if you do not have money, you still look smart during events. (Middle -aged woman living with HIV)

Many perceived this ‘medium’ figure to be ideal for middle-aged women, which conferred greater respectability in the community.

### Concerns about body weight

WLHIV participants appeared to be less concerned about DTG-related weight gain, which they regarded as a common feature of HIV treatment. Many of those reporting weight gain said it was “marginal”, “did not distract them from doing their normal work” and believed it would “not last long”. Weight gain was interpreted by some HCWs and WLHIV as an outcome of the ‘positive living’, which PLHIV are encouraged to embrace. They would only become concerned if they became “*extremely fat and face difficulty walking*” (Older woman living with HIV), or it resulted in a debilitating disease. Excessively fat women were noted to be widely stereotyped as ‘lazy’ and ‘useless’ and unable to perform daily activities expected of a wife, affecting their marriage and marriage potential.

Several women said they “*received a lot of compliments from friends and felt good”* (Younger woman living with HIV) for gaining weight after switching to DTG. Young women often differentiated two types of weight gain: one, which is desired, entail uniform increase across all parts of the body; and the other, which is disliked, involves excess fat at specific parts of the body such as abdomen, face, etc.,

I like to put on weight, but I do not want to get too fat to the extent of being shapeless. I want to gain bodyweight in a considerable way, one which is uniform. And not like one part of your body is big and other is small. (Younger woman living with HIV).

What most WLHIV dreaded was a rapid change in appearance that might draw attention to their HIV positive status. They noted that dramatic increases in weight would lead to suggestion that “*they are either using ARVs or family planning pills*” (Middle-aged woman living with HIV).

### Weight gain was underreported

Most women who gained weight had not reported it and lacked awareness that weight-gain was a potential side effect, “*I did not realize it. When you asked me, "did you gain body weight?" that is when I noticed because I am told that I am getting fatter and fatter.”* (Younger woman living with HIV). Those that recognised it said they either lived with it, hoping it would resolve by itself, or resorted to self-medication. They appeared to be most concerned with (and would only report) symptoms that left visible signs on their body or were debilitating:

It’s only fatness, it doesn’t kill. It is not stopping me from doing my work, it isn’t like I have developed rashes or body pains or organ failure. … I travel from very far to come to the hospital. So, I said "Maybe I should get a remedy that can help to reduce my body weight a bit" instead of going all the way to tell the Musawo<doctor> about it. (Middle-aged woman living with HIV)

This lack of awareness and of reporting had other negative health implications. In one reported incident, a WLHIV, who developed diabetes (allegedly from use of DTG-based regimen), developed diabetes-related complications leading to amputation because she failed to make the connection early so she could report to HCWs for a timely intervention.

### Effect of weight change on medication adherence

None of the WLHIV participants who experienced weight gain from DTG interrupted their medication, including those who were critical about gaining weight, “*I obviously continued… taking my drugs. Because gaining bodyweight is not a disease.”* (Younger woman living with HIV). Further, most had not checked the potential effects DTG drug on body weight before deciding to take it, nor would information about potential weight gain have affected their decision to take it. Some said weight gain from DTG use motivated them to improve medication adherence: *“I was happy it [DTG] made me to gain weight. So, I started taking it in full swing….”* (Younger woman living with HIV).

Women had limited understanding of health risks associated with weight-gain and tended to appreciate the benefits of ART a lot more than the risks associated with weight-gain. The potential for ART interruption leading to increased viral load and related deterioration of health was a common concern and a key reason woman did not stop treatment despite weight gain, as alluded by this participant: *“I do not mind about those side effects [ weight gain], I mind about my health. Because if I stop taking it, the viral load is going to increase, because ART is life* “(Middle-aged woman living with HIV).

However, many WLHIV participants suggested that their adherence would be affected if a DTG-based regimen made them slimmer. The few women who reported losing weight from DTG use feared they would be identified and stigmatised (as HIV positive), which affected their pill taking.

Everyone started to notice; saying 'eh! she is losing weight; what is she suffering from?' … that affected my attitude, I get discouraged, sometimes I will go without taking it (Younger woman living with HIV)

Some participants perceived weight loss to mean the drug is not effective or not suitable for them and therefore they should stop using it, as noted by this woman “*when you start using it [ ARV] and you see that you are losing weight it means that it is not good for you.”* (Younger woman living with HIV).

### Gaps in risk communication

WLHIV participants widely emphasised the importance of knowledge about side effects to reduce their anxieties and enhance adherence and timely reporting of side effects. Most said they were not informed about the full range of DTG side effects, especially those related to pregnancy, weight gain and diabetes. The lack of adequate information festered misconceptions, which may have undermined or promoted DTG uptake, including beliefs that “*DTG is going to cure us completely”* (Middle-aged woman living with HIV) and “*DTG acts as family planning*” (Younger woman living with HIV).

Information about possible side effects was provided in leaflets that were written in English and/or Luganda, often without an accompanying explanation from health workers. *“There is no information I was given. I was just called from the pharmacy and told, "you have been switched to this new drug, in case of any side-effect call us on this number”* (Middle-aged woman living with HIV). Several women reported that they could not read the leaflet. “*They gave me just a paper, but I can’t read”* (Middle-aged woman living with HIV). Those who could read said they found leaflets useful as they provided a reference to check against symptoms:

it was useful to me because it had those signs and symptoms. If it was not given to me, I would wonder 'what was happening to me'? … But when they gave it to me, I read it and know that "eh, this is the side-effect of the drug" and I stay strong [when I experienced the side effects]. I continue swallowing my drugs knowing that it will stop. (Middle-aged woman living with HIV).

Providers identified that the leaflets did not contain information about ‘newer’ side effects of DTG-based regimen such as hyperglycaemia and diabetes and suggested the need for updated leaflets that were concise, accessible using diagrams/pictures, and translated into common local languages.

Pre-treatment information on DTG-based regimen was provided in group health talks by peer educators. Both the health care provider and women identified that such talks were not targeted and lacked opportunity and privacy to ask personal questions and learn about treatment, as suggested by this woman:

there wasn't so much one-on-one as in someone giving you details about the drug…, maybe that's why I did not get to understand the side effects. (Older woman living with HIV)

Some providers were not sufficiently transparent about potential side effects of DTG-based regimen. Programmatic targets for facilities to transition people to DTG-based regimen were noted to have resulted in practices that undermined patients’ autonomy over their treatment. Many HCWs described being cautious so as not to discourage patients from using the drug. Instead, they downplayed side effects while emphasising benefits.

I tell them, but more of the benefits not really the risks. … I tell them with caution of the challenges, we say “the side effects are not so common”. … we say to them please return to the Musawo<doctor> if you experience any problem. (Clinician)

Women also alluded to lacking support at home or with friends with whom they could discuss and understand their weight gain. Those who had not disclosed their HIV status regarded any side effect as a secret and rarely discussed symptoms with other people to confirm or discount the cause. Others failed to disclose their HIV positive status and treatment when they consulted with health workers outside of the HIV specialist clinics.

### Barriers to monitoring and treating weight gain and related complications

Both WLHIV and health care providers in HIV clinics pointed to challenges in recognising and monitoring weight gain and associated co-morbidities. While most facilities had scales, recording of patient weight was inconsistent. The lack of equipment and resources, especially in lower-level facilities, was widely identified, for example, frequent stock-out of glucose test kits meant health workers were not able to check for baseline diabetes before initiation, as mandated by the treatment guidelines, nor monitor the blood sugar level of patients.

at times we may go without kits for testing random blood sugar, so we’re not able to get to the bottom of their weight gain. (Nurse)

Health care providers described having to refer patients who presented with weight gain to higher level hospitals and private providers, which brought financial cost to patients. Women reported having to buy drugs from out-of-pocket to manage side effects experienced from DTG use, *“When I had the diabetes the doctor prescribed medicine for me worth shs.200,000, shs.150,000, shs.300,000. I could not afford it. I waited for about 2 weeks until my relatives bought it for me. …Whenever the dose is getting finished, I get worried”* (Older woman living with HIV).

Additional commodity challenges were related to stock-out of alternative efavirenz-based regimens, which constrained health care providers ability to switch patients with weight gain issues, “*we have been having challenges of stock outs of TLE [alternative ART regimen], which makes it hard for us to switch those who are not responding well to DTG”* (Clinician).

Further, low staff ratio and related high workload were noted to affect effective vigilance for weight gain issues. Some providers said they were unable to spend as much time as the would have liked with HIV patients that led to them missing important side effects.

we tend to ask the patient ‘what is your illness today?' But forgetting—'how was the drug? … what have you experienced?' … problem is sometimes you only have 4 nurses looking after a clinic of 200 patients, so it is just impossible to do this for everyone …. (Nurse)

There was no national or local guidance on how to deal with DTG-associated weight gain and related health problems. Providers noted a lack of clarity about what level of weight gain they should be concerned about, given that some weight gain may be positive and suggest a return to health. Information on how to manage significant weight gain and associated co-morbidities are limited in current national treatment guidelines, resulting in management inconsistencies.

We don’t have a protocol that we are going to follow in case someone comes with this or that level of sugars. So, I will come with my ideas, we know how to manage hyperglycaemia, but we have different ideas, so, I could choose to withdraw the drug and … someone else will chose to start them on insulin immediately, so if we could have seniors’ intervention or a consistent protocol, … I think it would be better. (Clinician)

Patients recognising and presenting with ‘minor’ weight gain to HIV clinics were given nutritional education and encouragement from health care providers to modify their lifestyle, including “*making changes to their diet*”, “*eating less high protein food*”, and “*engaging in regular exercise*”. Such lifestyle modification advice was unappealing to several WLHIV who complained about lack of food options. Lactating mothers were especially concerned they would struggle to feed their babies if they were to make dietary modifications. There was mixed response to participating in a weight loss programme, with many women suggesting they would be reluctant to join, citing fear of growing thin, old age, and lack of strength. It was widely believed weight loss programmes are meant for the rich “*who become fat through eating good food and not for the poor”* (Middle-aged woman living with HIV). Several suggested they would only participate if it was made mandatory or recommended by their doctors: *“I would join If the doctors recommended it. But if it’s my choice to make, I would not join”* (Younger woman living with HIV).

## Discussion

Overall, our findings indicate an aversion to thin body size and a social benefit to gaining a moderate to high level of weight among WLHIV taking DTG in Uganda. DTG-associated weight gain did not appear to affect women’s adherence to treatment, whereas treatment-associated weight loss did impact adherence. Clinical management and pharmacovigilance of DTG-related weight gain issues are constrained by womens’ limited knowledge of what to expect and of the health risks associated with being overweight in the context of treated HIV infection. While this was a major source of worry for health care providers, it was less of a concern for WLHIV, often leading to incongruent treatment expectations and a low uptake of weight loss interventions among WLHIV. The lack of clear communication, access to diagnostic equipment and resources to investigate weight gain and limited clinical guidance emerged as vital health system factors.

The aversion to thinness observed in our study has been demonstrated among other PLHIV[35, 36]. In the current study, WLHIV preference for a larger body size was related to the need to conceal their HIV positive status (41). Studies have suggested that HIV positive status may have a potential buffering effect against perceived weight stigma experiences, in part, due to historical links between HIV infection and low body weight [37, 38]. Thinness is commonly perceived in African societies to be associated with poverty and disease[39] and weight loss was perceived to lead to people suspecting HIV. Persson noted this in relation to PLHIV with lipoatrophy (a condition with facial wasting and central bodily fat accumulation linked to early antiretrovirals) perceiving that HIV is ‘written on the face’, inviting social gaze, and making it difficult to maintain secrecy about HIV status[40]. The desire to keep one’s HIV status secret is critical in our study context where sero-disclosure is low and carries negative consequences, such as stigma, discrimination, and intimate partner violence[41, 42]. The body represents the medium through which a positive HIV status is discernible to society and changes in appearance are a trigger for social and personal stigmatisation[43]. Following HIV diagnosis, women may become more image conscious and experience heightened need to be attractive to boost their self-confidence and crowd out the possibility of people finding out about their diagnosis. They do so by aspiring to cultural ideals of beauty, which in the study context was moderate to large body size.

Our findings underscore the need for health professionals to take serious account of body size perceptions in shaping women’s body image, social interaction, and psychosocial wellbeing[40] if they want to improve HIV treatment outcomes. Gibson posits that WLHIV construct complex and fluid relationships with their bodies shaped by cultural discourses of the female body, physical changes to the body following diagnosis, and HIV-related stigma[14]. Even when they had not lost weight, women in our study were constantly anxious that their bodies may show symptoms of HIV infection and aspired to gain weight to look attractive.

The perceived weight gain associated with DTG-based regimen is consistent with the work of Nabitaka and colleagues in Uganda [44] (48) and corroborates other studies [10, 45, 46]. Current health promotion approach in Ugandan facilities encourages patients with weight gain on DTG to reduce weight through dietary and lifestyle modifications. This may be ineffective if WLHIV are content with their body size or desire a large body size to thrive. Routine discussion about body size and health should be instituted to enable providers to discuss preferences and concerns and to develop intervention plans that are contextually relevant and tailored to the patient [36, 47]. Further, body image perceptions and experiences of PLHIV are dynamic and changeable [48], and thus require ongoing attention and discussion. Previous observations, mostly made in the context of lipodystrophy, have suggested that weight gain may reduce adherence to ART [44]. Contrary to this, our data indicated that moderate to high weight gain does not reduce adherence, and in some cases motivated greater adherence among women. Women’s limited knowledge of the actual health risks led many to underplay these risks and may have distorted their body size preferences making them less likely to report weight gain.

Several practical recommendations for healthcare providers arise and are outlined in Table 3. Further, there is a lack of sufficient guidance on how to manage weight gain and related complications of DTG. Although the current Ugandan ART guidelines mandate regular weight and blood glucose monitoring for patients on DTG-based regimens, this has been hindered by general deficiencies in the laboratory infrastructure and commodity stock availability [22]. Improved funding for diagnostic equipment and laboratory infrastructure is critical to supporting early detection of metabolic diseases associated with weight gain. There is the need for clear protocol for clinical management of DTG-associated weight gain and related health issues. The findings point to synergistic benefits from integration of HIV services with the management of non-communicable diseases such as diabetes and hypertension [35], and further suggest the need for stronger set up for management of chronic disease in general.

**Table 3:** Practical recommendations for healthcare providers

Regular discussion with patients about their own appearance and body weight preferences to inform treatment and weight management plans
Active vigilance and monitor weight gain and potential development of co-morbidities in DTG patients, including regular weighing and recording of weight
Baseline screening patients during ART initiation to identify those with high risks for overweight and diabetes for targeted support
Information about DTG side effects should be provided through leaflet along with verbal explanation by HCWs; regular update of the leaflets with new side effects discovered
Treatment education and counselling should be conducted through group sessions along with one-to-one engagement with patients

## Limitations

The cross-sectional design of the study only offered a snapshot of the perceptions of WLHIV about their body image and experience of DTG-related weight gain and therefore limits our ability to make causal inferences. A longitudinal study would add greater insight on body image perceptions and the dynamic impacts of weight gain on adherence behaviour over time, particularly as weight gain may not be clinically problematic over the short term. The purposive sampling approach, the focus on women and the small sample size provided an in-depth exploration but may limit the generalisation of the results to broader population of PLHIV and different settings. Weight gain issues are also pertinent to men, and a similar study may be needed to understand men’s perspectives. Some WLHIV participants had not experienced weight gain, nor were they aware DTG was associated with weight gain, making it difficult to explore their lived experience. Given the personal nature of body image it is possible social desirability bias and discomfort talking about personal issues impeded the depth and reliability of some of the responses.

## Conclusion

This study demonstrates the significance of large body-size in enhancing positive body image, preventing unintended sero-disclosure and related stigma, and promoting psychosocial wellbeing among WLHIV in Uganda. While weight gain from DTG-use is widely recognized as a side effect by WLHIV and HWs, weight gain may be welcome by WLHIV and less likely to reduce medication adherence but could lead to underreporting and greater risk of cardiovascular and metabolic diseases among WLHIV. Regular and proactive monitoring for weight gain and potential development of co-morbidities in patients initiated on DTG-based regimens is recommended. Providers should account for the perceptions of WLHIV about their own appearance and body weight preferences to ensure optimal treatment and positive health outcomes. Future research will benefit from an exploration of men’s perspectives of weight gain from HIV treatment as well as a longitudinal assessment of the effects of weight gain on adherence behaviour among both men and women.

## Supplementary Information

**Additional file 1.** **Appendix 1**: Semi-structured topic guides for used for interviewing women living with HIV and healthcare workers

**Additional file 2.** **Appendix 2**: Completed COREQ checklist for manuscript

## Data Availability

Data are available from the corresponding author on request**.**

## Abbreviations

3TC: Lamivudine
ART: Antiretroviral therapy
DTG: Dolutegravir
EFV: Efavirenz
HCW: Healthcare workers
HIV: Human immunodeficiency virus
PLHIV: People living with HIV
RA: Research assistant
WHO: World Health Organization
WLHIV: Women living with HIV
