# Supplementary material for: “It’s only fatness, it doesn’t kill”: a qualitative study on perceptions of weight gain from use of dolutegravir-based regimens in women living with HIV in Uganda
Source: BMC Womens Health. 2022 Jun 21;22:246. doi: 10.1186/s12905-022-01814-x (PMC9210809; doi:10.1186/s12905-022-01814-x)
Supplement: Supplementary file 1 — Additional file 1. Appendix 1: Semi-structured topic guides for used for interviewing women living with HIV and healthcare workers [file 12905_2022_1814_MOESM1_ESM.docx]

**Appendix 1: Semi-structured topic guides for used for interviewing women living with HIV and healthcare workers**

**Women living with HIV**

1. Please tell me a little about your experience on ART

- How long have you been on it?
- What drugs have you used in the past? How did they treat you?
- What ARV drugs are you using now?
- How long have you been/were you on DTG?

**Risk communication**

1. What do you understand are the benefits and risks (side effects) of DTG?

- Describe the benefits
- Describe the risks (side effects)
- Probe about risks in pregnancy (e.g., neural tube defect)
- What would you do if you suffered side effects?

1. What kind of information was given to you when you were being initiated/switched onto DTG?

- Were you informed about the risks (side effects) of the drug? what did they say?
- Were you informed about the risks in pregnancy? what did they say?
- Were you informed about what to do if you suffered any side effects? Explain
- Who gave you that that information?
- Was the information useful? Was it sufficient? What else did you wanted them to explain to you?

1. How was information about DTG given/delivered to you by the healthcare professional?

- Probe if delivered orally or leaflet, and what language used
- Probe if given the chance to ask questions and if all questions were answered
- What did you like about how the information was communicated to you?
- What did you not like about how the information was communicated to you?
- What changes would you like to be made to how information about ART is communicated?

1. How did information about the risks (side effects) associated with DTG make you feel about the drug?

**Experience of side effects**

1. Tell me about your experience of using DTG. Did you think that the drug (DTG) may have caused you any problems?

- Please describe any side affects you may have experienced from using the drug
- What made you to think that those side effects were associated with DTG? Did you confirm the suspected side effects with any information sources? Explain
- How did the symptoms/side effects make you feel about the drug (DTG)?

1. How did the symptoms (side effect) affect the way you took your medication? Explain.

- Probe about effect on medication adherence (e.g. pill taking)

1. How else did the symptoms/side effect affect other aspects of your daily life/lifestyle?

- Probe about household work, economic, social and psychological/emotional effects

1. How did you deal with the symptoms (side effect)?

- Did you try to treat the side effects yourself through medication, natural remedies or lifestyle change? explain
- Did you get help from any healthcare professionals? Who? What did they do?
- How did you feel about the help you received from the health professional? Were you satisfied with the support you received? what else did you want them to do?

1. What support do you and other women need to help you better deal with DTG related side effects?

- What changes would you like to see made to how HCWs support with side effects

**Perceptions about body weight**

1. What are your preferences about body weight?

- Do you prefer big or slender? why?
- Probe preference during: Pregnancy, Breastfeeding, Living with HIV
- What benefits and risks do you associate with big and slender body weight among women? (also probe in relation social benefits and risks – e.g. stigma)

1. How do you feel about your body weight now?

- Have you ever been concerned about your body weight? When? How?
- Has your HIV diagnosis changed your body weight preference? Why?
- What does your partner (if applicable) think about your body weight?
- For adolescents especially those of school-going age probe for effect of peer pressure on weight changes

1. What do people in your community expect of the body weight of women?

- What body weight do they expect of: Pregnant women, HIV positive women, Lactating mothers? Explain
- What do your peers think about their body weight? What do they prefer? Why?

1. Is your body weight affecting your wellbeing in anyway? How?

- Probe effect on social life, lifestyle, participation in economic activities, emotional/psychological wellbeing, additional costs incurred in buying clothes, health concerns and buying food

1. How important is body weight to you in your decision-making about the medication you use?

- Do you check to see if the drugs you use affect your body weight before deciding to use it? How?
- Will your decision about taking the drug change if you were told it makes you fatter or slimmer? How?
- Will you stop taking your medication if you realised that it is making you fatter or slimmer? How?

1. [*One of the side effects of DTG is body weight*]. Did your body weight change while using DTG? How?

- [If YES] How did that make you feel about the drug? What did you do in response? Did it make you change how you use the drug (adherence)?
- [If NOT] How would you feel if DTG were to make you gain weight? What would you do in response in terms of your treatment?

1. Would you be willing to join a fitness/nutritional program if it was in place to help you lose weight?

**Healthcare workers**

1. Please tell me a little about your experiences of prescribing DTG-based regimen

- What do you think about the switch to DTG? Was it necessary?

1. Have you encountered any challenges so far in relation to prescribing DTG to patients? Please explain.

**Risk communication and response to side effects**

1. What kind of information do you give to patients when initiating/transitioning them onto DTG?

- Probe about whether they inform patients about benefits, risks/side effects, and what to do if they suffered side effects.

1. How do you educate/communicate to women about the benefits and risks/side effects associated with DTG?

- What challenges do you face in educating/communicating to women about their ART treatment?
- What changes would you like to see in the way that women are educated/informed about their treatment risks/side effects?

1. Are patients (women) given a chance to influence decisions about their treatment? If so how?

- What do you think about the suggestion that women should be given an informed choice about DTG use?
- How do you currently provide women with an informed choice?
- What challenges do you face in giving women an informed choice?
- Are there any changes you would like to suggest about giving women an informed choice?

1. Tell me about your experiences of dealing with patients who have suffered side effects related to DTG use?

- What side effects have been reported to you?
- How common are these?
- Are there any that are peculiar to women?

1. What support do you give to patients (women) who report side effects from DTG?

- How do you feel about this support/response - is it adequate?
- Are there things you think should be done differently?

1. Do you normally have to report the side effects to the National Drug Authority and/or other organisations?

- How do you feel about this reporting system?
- What challenges do you face with it?
- What changes would you suggest should be made to improve the process/system?

1. As far as you are aware do the side effects affect the way patients take their DTG medication? How?

- Probe potential effect of the side effects on medication adherence

1. Do the side effects impact on other aspects of patient’s life? How?
2. Now that there is a switch to DTG regimen what do you think are some of the things that are likely to affect ART adherence among women?

**Body weight**

*[I would like us to discuss a bit more about perceptions and effects of body weight among women.]*

1. What are women’s preferences about their body weight?

- Fatter or slimmer? Please explain
- Probe preferences in relation to: Pregnant women, HIV positive women, Lactating mothers

1. How important is body weight in women’s decision-making about their HIV treatment?

- Do they check to see if the drugs affect their body weight before deciding to use it? How?
- Do women change their decisions about a drug if you were told it will make them fatter or slimmer? How?
- Do women stop taking their ARV drugs if it makes them fatter or slimmer? How?

1. Do you think DTG causes weight gain? Explain

- Have you had any cases of weight gain among DTG patients before? If yes, explain.
- What did the patient do?
- What did you do in response?
